# Investigating zeta‐cypermethrin resistance stability in California *Drosophila suzukii* populations

**DOI:** 10.1002/ps.70760

**Published:** 2026-03-26

**Authors:** Nicolas Buck, Fatemeh Ganjisaffar, Frank G. Zalom

**Affiliations:** ^1^ Department of Entomology and Nematology, One Shields Ave University of California Davis CA USA

**Keywords:** dose–response, insecticide, LC_50_, pyrethroid, spotted‐wing drosophila

## Abstract

**BACKGROUND:**

*Drosophila suzukii* (Matsumura) causes serious damage to soft and stone fruit globally, resulting in substantial economic losses. Due to its unique ability to oviposit in and degrade ripe fruit, management programs rely primarily on insecticides to minimize pest pressure. While insecticide resistance has been reported in *D. suzukii* populations, the stability of this resistance remains unknown. We assessed resistance stability to the pyrethroid insecticide zeta‐cypermethrin in a field‐collected *D. suzukii* population under continuous laboratory selection pressure over multiple generations and after the selection pressure was removed. We further assessed resistance stability following a population bottleneck resulting from applications of a greater concentration of the insecticide following initial laboratory selection.

**RESULTS:**

Following termination of continuous zeta‐cypermethrin laboratory selection for 20 generations, the LC_50_ of the field‐collected *D. suzukii* population remained stable for an additional 16 generations. A second field‐collected population initially selected in the laboratory then subjected to a greater insecticide concentration to create a population bottleneck then followed by continued zeta‐cypermethrin exposure after a recovery period resulted in increased resistance as indicated by LC_50_ values.

**CONCLUSION:**

Field derived resistance to a commonly used pyrethroid insecticide in *D. suzukii* can persist post‐selection and following a population bottleneck in a laboratory environment. These are the first studies to assess insecticide resistance stability in this economically important pest, and results warrant consideration for pest management programs. © 2026 The Author(s). *Pest Management Science* published by John Wiley & Sons Ltd on behalf of Society of Chemical Industry.

## Graphical abstract

After continuous selection and removal of selection pressure in spotted‐wing Drosophila, zeta‐cypermethrin resistance remained stable over several generations. Following a population bottleneck, zeta‐cypermethrin resistance increased without selection, highlighting the importance of insecticide rotation.

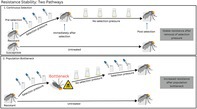

## 1 INTRODUCTION

Spotted Wing Drosophila (SWD), *Drosophila suzukii* (Matsumura) is a pest of soft and stone fruit that poses an economic risk to crops globally. Adult females use a sharply serrated ovipositor to lay eggs in the epicarp of the fruit, and the subsequent larval feeding not only damages the fruit flesh but allows a pathway for pathogens to further degrade the quality of the fruit.^1^, ^2^, ^3^, ^4^, ^5^ Unlike other pests of soft fruit that lay eggs in rotten or dropped fruit, *D. suzukii* oviposit in ripe fruit often making detection difficult in the field during harvest.^6^ *Drosophila suzukii* has a wide host range, targeting fruits with thin skin such as blueberry, strawberry, raspberry, grape, and cherry.^7^ In a study conducted across Italy, Switzerland and the Netherlands, *D. suzukii* were recorded emerging from 84 different plant species, highlighting the wide host range of the pest.^8^ The first reports of *D. suzukii* in North America were in California in 2008. In Europe, the first records were in Spain in 2008, and by 2012, it had spread across most of Europe.^3^, ^9^, ^10^ *Drosophila suzukii* also have quick generational life cycles and undergo seasonal variation, with winter morphs that enter diapause being more cold tolerant.^11^, ^12^ Their rapid spread, wide host range, short generation time, and overwintering capacity have posed complications for pest management strategies across many cropping systems.

Due to the widespread use of chemical insecticides, both organic and conventional, to reduce *D. suzukii* pressure on fruit farms, insecticide resistance has been reported within the last few years. One study using a larval bioassay identified the ability for *D. suzukii* to rapidly develop insecticide resistance in the commonly used insecticides malathion and spinosad under laboratory conditions.^13^ However, and more crucially, insecticide resistance has been documented in field‐caught populations. Flies from Watsonville, California were shown to have spinosad LC_50_ values (the lethal concentration required to kill 50% of the sampled population) 4–7 times greater than the nearby untreated sampling location and 11–24 times higher than baseline susceptible populations indicating a significant increase in insecticide resistance.^10^ The first field‐derived pyrethroid‐resistant populations of *D. suzukii* were recorded from four different conventionally‐managed sites in California in 2022, however bioassays showing increased tolerance as the field season progressed in two of these sites indicating a build‐up of tolerance correlating with more exposure of the insecticide in the local populations.^14^ One study has even found resistance to the neonicotinoid insecticide imidacloprid in Brazil, with the key driver likely being the activity of certain detoxification enzymes.^15^ In the closely‐related *Drosophila melanogaster* Meigen (Diptera: Drosophilidae), field‐collected populations were shown to have zeta‐cypermethrin, acetamiprid and malathion resistance in New York.^16^, ^17^ Clearly, insecticide resistance development in *D. suzukii* across widely used insecticide classes should be a concern for small fruit and stone fruit growers throughout its established geographic range.

Other research has also identified fitness costs associated with insecticide resistance that could be utilized in pest management programs if resistance is detected in field populations.^18^, ^19^, ^20^ These fitness costs could affect resistance stability, if populations are producing fewer progeny each generation which could then become a focal point for management practices.

While research has identified insecticide resistance in *D. suzukii* to a variety of commonly used insecticides, no study has assessed the stability of the resistance, through a population bottleneck, as with our field‐derived resistant populations tested. We believe these two scenarios are likely to occur on soft fruit farms due to insecticide rotation and seasonal population dynamics. In this study, we use field‐caught California populations of *D. suzukii* with already‐established baseline resistance levels and maintain a selection pressure for several generations, assessing the resistance levels before, during and after removing the selection. We assess the stability of resistance and in a separate population assess resistance stability following a population bottleneck.

## 2 MATERIALS AND METHODS

### 2.1 Continuous selection study

The source of *D. suzukii* for this 2019–2023 study was from caneberries near Castroville, Monterey Co., California by capturing live adults using plastic McPhail traps (Great Lakes IPM, Inc., Vestaburg, MI, USA) containing a 20‐mL mixture of 7 g yeast, 113 g sugar, and 355 mL water at a site designated ‘MO’. The raspberries in that region were trellised and grown in three‐row hoop tunnels as is typical for that region and were regularly treated with pyrethrin. However, the neighboring site and all fields surrounding them were regularly treated with pyrethroids, so exposure to the insecticide zeta‐cypermethrin was common, indicating continuous on‐field resistance selection. At each site, five baited traps were deployed in the middle row of three selected hoops. Traps were modified with a mesh barrier to prevent *D. suzukii* from drowning in the liquid lure. After 24 h, traps were transported to the University of California, Davis and chilled at 4 °C for 5–10 min to facilitate the aspiration of *D. suzukii*. Collected flies (15 males and 15 females) were transferred into 177.4 mL (6 oz) plastic Fisherbrand Drosophila bottles (Fisher Scientific, Inc., Portsmouth, NH) containing Bloomington standard *Drosophila* cornmeal diet and transferred to new diet bottles every 6 days.

MO flies exhibited high survival when exposed to the LC_90_ × 8 discriminating dose (6.89 ppm) of zeta‐cypermethrin,^21^ indicating the presence of resistance. Baseline susceptibility to zeta‐cypermethrin was assessed in the already‐resistant F1 generation adults using a dose–response bioassay at concentrations of 1, 3, 6, 10, and 20 ppm (8–9 replicates per concentration), establishing the susceptibility profile of the MO population prior to selection. This was carried out by coating the inside of 20 mL glass scintillation vials (Fisher Scientific, Portsmouth, NH, USA) with the respective zeta‐cypermethrin concentrations and letting them dry with lids off in a fume hood for 24 h. Five male and five female adults (3–5 days post‐eclosion) were then loaded into the vials with an aspirator and covered with treated caps. The treated vials were kept on their side in a tray on a lab bench by a window providing natural light, after which mortality per sex was assessed as dead (motionless or unable to right themselves when on their back) or alive.

Selection for zeta‐cypermethrin resistance was conducted from generations F1 through F18 using the bottle bioassay method described in a different study.^13^ For each generation, groups of 30 mated females (identified by a sharply serrated ovipositor and no wing spots, 3–5 days post‐eclosion) were transferred into the plastic Drosophila stock bottles containing the aforementioned cornmeal diet to oviposit for 4 days. Females were then removed and transferred to fresh bottles for the next replicate. Immediately after female removal, 400 μL of 13 ppm zeta‐cypermethrin solution (previously determined as the LC_90_x2 concentration for resistant larvae by Gress and Zalom, 2022)^13^ dissolved in acetone was pipetted onto the diet surface and distributed evenly by swirling the bottle. This ensured larval exposure both through external contact and internal ingestion during feeding. Control bottles received 400 μL of acetone only. Bottles were replugged and maintained at 23 °C. Adult emergence was monitored daily for up to 18 days post‐treatment, with all emerged flies counted. Each subsequent generation was established using progeny that successfully emerged from insecticide‐treated bottles, maintaining continuous selection pressure. Selection was paused at F19 to increase population size in preparation for bioassays at F20 and F35.

Post‐selection susceptibility was assessed using dose–response bioassays at F20 (1, 3, 6, 6.89, 10, 20, 36, 54, 72, 80, and 90 ppm, 10 replicates per concentration) and again at F35 (6.89, 10, 20, 36, 48, 54, 72, and 90 ppm, 10 replicates per concentration) using the aforementioned dose–response bioassay protocol. All bioassays included untreated controls (0 ppm) to assess baseline mortality. Data were aggregated by summing mortality counts across replicates for each unique combination of dose, sex, and population.

A susceptible reference population (Wolfskill) was obtained by similarly capturing live adults from an untreated mixed fruit orchard at the University of California, Davis Wolfskill Experimental Orchards near Winters, Solano Co., CA (roughly 185 km north of the study site). Wolfskill flies were previously established as a susceptible control in a different study,^14^ exhibiting no survival when tested against the discriminating dose of zeta‐cypermethrin (6.89 ppm). In addition, because whole genome sequencing indicates that California *D. suzukii* populations lack distinct genetic structure,^22^ the Wolfskill colony was considered an appropriate untreated control. Both colonies were maintained in a walk‐in chamber at 23 ± 1 °C, 55–65% RH, with 14:10 (L:D) h photoperiod. A dose–response bioassay was conducted using concentrations of 0, 0.1, 02, 0.5, 1, 2, 4, and 6.89 ppm to determine susceptibility of Wolfskill flies using the same protocol.

### 2.2 Population bottleneck study

The source of *D. suzukii* for this 2024 study were infested cherries collected from a conventionally‐managed farm near Morgan Hill, Santa Clara Co., California, sent to our lab at University of California, Davis and emerging flies were reared on Drosophila diet in the same manner as in the continuous selection study. This population is called ‘M. East’. Baseline susceptibility to zeta‐cypermethrin was determined for the F2 and F8 generations adults of M. East using the dose–response vial bioassay at concentrations of 6.89, 30, 60, 120 and 180 ppm. This dose–response bioassay method was the same as in the previous study, except the bottles were kept on a bench top in the laboratory at room temperature (~23 °C) with windows providing natural light as opposed to being kept in a climate‐controlled chamber. Previous bioassays for a separate project in our lab were carried out using the same methodology in both the climate‐controlled walk‐in chamber and the lab bench, and no difference in mortality was observed between the two when using the same insecticide concentration on the same generation of a population.

After completing the dose–response bioassay, the population was maintained for six generations to increase adult numbers for the study which began at F8. A discriminating dose of 15 ppm (equivalent to half of the label rate concentration when applied at 935.4 L ha^−1^) was carried out using 10 vials of F8 adults (sex ratio of 5 females: 5 males) to ensure zeta‐cypermethrin tolerance was still present. Once confirmed, the selection treatment began.

The selection treatment was implemented using a similar method to the dose–response bioassay wherein five male and five female adult *D. suzukii* were aspirated into already‐treated vials (of 15 ppm zeta‐cypermethrin) and left for 6 h after which mortality for both sexes was assessed. However, unlike the dose–response bioassay, flies that were alive at the time of mortality assessment were then deposited into fresh diet bottles to mate. Once pupae were visible, the adults from these bottles were placed into fresh diet bottles to continue oviposition. The next round of treatment was carried out within a 5‐day window after the first adult had emerged (these were the offspring of the previously treated adults). Replicates (number of vials per round of treatment) varied between 6 and 16 due to the differing availability of eclosed adult flies per treatment. This selection treatment was carried out on adults every generation from F8 to F10 at 15 ppm, and from F11 to F12 at 30 ppm (equivalent to the label rate concentration when applied at 935.4 L ha^−1^). Our intent was to use a selection concentration that resulted in ~70% survival for generations 8–10 and then increase the concentration beginning in generation 11 to restrict survival without eradicating the population. The dose response carried out on the F1 generation showed 56% survival at 15 ppm, and so the population size was increased to prepare for potential losses at the start of the study. Once minimal mortality (~15%) was observed at generation 10, a 30 ppm concentration was then used to challenge generations F11 and F12, and then selection was suspended after F12 to allow the population to recover. Selection was then resumed from F19 to F21 at 15 ppm and the study terminated. Mortality was assessed using the vial bioassay as previously described at every round of treatment. A sub‐group of the M. East population was kept in the same conditions as the main treated group; however, the only difference was that it was not exposed to zeta‐cypermethrin. Every round of treatment, five vials coated only with acetone (hereafter referred to as 0 ppm) were loaded with five males and five females of this control group to compare with the treated group treated with 15 ppm of zeta‐cypermethrin.

### 2.3 Statistical analysis

#### 2.3.1 Continuous selection study

Dose–response relationships were analyzed using two‐parameter log‐logistic (LL.2) regression models fitted with binomial error distributions implemented in the drc package in R. We fit these log‐logistic models separately for males and females where the dose–response function was modelled as a log‐logistic curve with population‐specific slope and LC_50_ parameters. Model adequacy was assessed using goodness‐of‐fit Chi‐square tests. Significant lack‐of‐fit (*P* < 0.05) indicated overdispersion (variance in the data exceeds that expected from a simple binomial model). For females, the overdispersion factor was 2.45 (χ^2^ = 53.82, df = 22, *P* < 0.001); for males, it was 3.33 (χ^2^ = 73.28, df = 22, *P* < 0.001). To properly account for this overdispersion, we calculated confidence intervals for LC_50_ and LC_90_ estimates (doses causing 50% and 90% mortality, respectively) using bootstrap resampling with 999 iterations, stratified by population. Bootstrap confidence intervals (CI) were calculated using the percentile method at the 95% confidence level with the ‘boot’ package in R. Because the log‐logistic regression models do not account for overdispersion, we also assessed differences by examining whether bootstrap 95% CIs overlapped. Differences were considered significant if the original log‐logistic regression model gave a significant *P*‐value as well as if the 95% CIs for this comparison did not overlap. LC_90_ values were calculated but ultimately excluded from the results due to insufficient high dose data that resulted in extremely large standard errors and failure of bootstrap iterations.

#### 2.3.2 Population bottleneck

We analyzed mortality data using generalized linear models (GLMs) with binomial error distribution and logit link function. The response variable was specified as a two‐column matrix of dead and alive females. Fixed effects included experimental phase (pre‐bottleneck *vs*. post‐recovery), insecticide concentration (0 or 15 ppm), and their interaction. Pre‐bottleneck was categorized as generations 8, 9 and 10 that were treated with 15 ppm (this excluded generations 11 and 12 that were treated with 30 ppm), and post‐recovery was categorized as generations 19, 20 and 21 that were again treated with 15 ppm.

Initial model fitting revealed quasi‐complete separation (very low mortality in some treatment groups) in the data, a known problem in logistic regression when outcomes are highly predictable.^23^ To address this, we used Firth's penalized likelihood method^24^ using the brglm2 package, which adds a penalty term to the likelihood function to produce finite, less biased parameter estimates.

We then used estimated marginal means (EMMs) to compare: (i) mortality between pre‐bottleneck (generations 8–10) and post‐recovery (19–21) phases at each concentration (0 and 15 ppm) and (ii) dose–response slopes between phases using the emtrends function. All estimates were back‐transformed from the logit scale to probabilities with 95% confidence intervals.

## 3 RESULTS

### 3.1 Continuous selection

**Figure 1:**
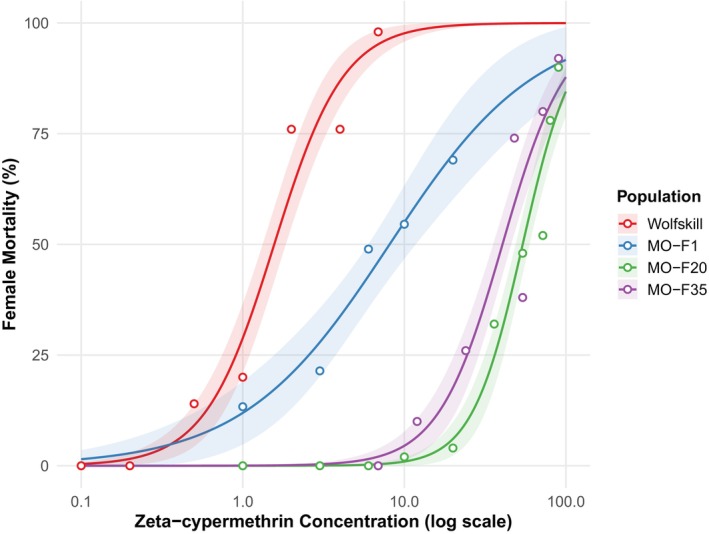
Dose–response curves for female *Drosophila suzukii* exposed to zeta‐cypermethrin across a range of concentrations for each experimental group: Wolfskill, and generations 1 (preselection), 20 (immediately after selection termination) and 35 (post selection) of the ‘MO’ population. Solid lines represent fitted two‐parameter log‐logistic models, with shaded regions indicating 95% confidence intervals (a separate metric from the 95% confidence intervals in the bootstrap resampling). Open circles show observed mortality proportions for individual dose‐population combinations. The x‐axis presents insecticide concentration on a logarithmic scale (ppm), while the y‐axis shows percent mortality.

The LC_50_s for both the susceptible population from the untreated site (Wolfskill) and the first generation of the tolerant field population (MO‐F1 ‐ preselection) obtained prior to initiation of continuous treatment served as baseline resistance values for each population for comparisons to the continuously‐treated (MO‐F20 – immediately after selection termination) population and the same population after treatments were withheld (MO‐F35 ‐post selection). Dose–response curves for female flies revealed clear differences among populations across the concentration range tested (Fig. 1). The Wolfskill population was the most susceptible to zeta‐cypermethrin, with an LC_50_ of 1.57 ppm (95% CI: 1.17–2.19, Table 1, Fig. 1). The MO‐F1 population exhibited significantly greater tolerance with an LC_50_ of 8.11 ppm (95% CI: 6.38–14.48), representing a 5.2‐fold increase in survival relative to Wolfskill. The MO‐F20 and MO‐F35 populations displayed the greatest resistance levels, with LC_50_ values of 54.03 ppm (95%CI: 46.84–65.38) and 40.51 ppm (95% CI: 31.73–58.37), respectively, corresponding to 34.5‐fold and 25.8‐fold increase in survival compared to the susceptible baseline Wolfskill. While the 95% CI for MO‐F20 and MO‐F35 overlap, indicating no significant difference in LC_50_ values between the two, they are both significantly greater than Wolfskill and MO‐F1, indicating a significant increase in resistance after continuous selection with no reduction in mortality even after treatment was withheld.

**Table 1:** Examination of bootstrap 95% confidence intervals by population group for females and males

Sex	Population group	Estimate	Standard error	Lower 95 CI	Upper 95 CI
Female	MO‐F1	8.11	6.417	6.38	14.48
Female	MO‐F20	54.03	4.703	46.84	65.38
Female	MO‐F35	40.51	7.507	31.73	58.37
Female	Wolfskill	1.57	0.347	1.17	2.19
Male	MO‐F1	2.821	57.356	1.262	3.63
Male	MO‐F20	29.668	2.516	24.912	34.21
Male	MO‐F35	18.315	529.712	9.098	140.72
Male	Wolfskill	0.644	0.224	0.506	1.34

**Figure 2:**
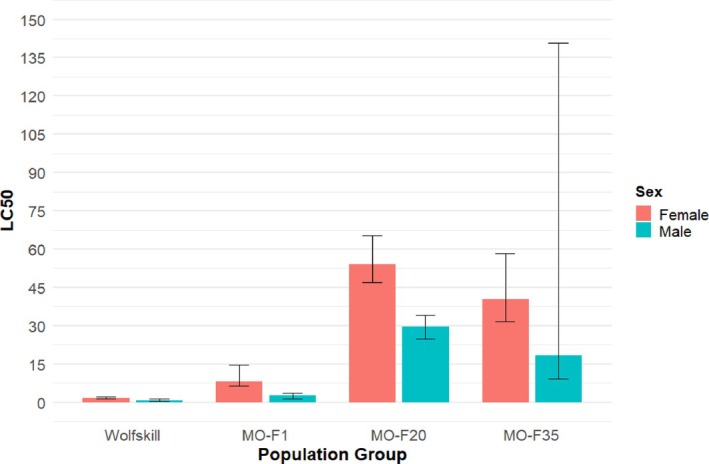
LC_50_ (lethal concentration to kill 50% of the population) and standard error for each experimental group: Wolfskill, and generations 1 (preselection), 20 (immediately after selection termination) and 35 (post selection) of the ‘MO’ population. For each experimental group female mortality is on the left, and male mortality on the right, both with error bars.

For males, the Wolfskill population and MO‐F1 were the most susceptible population groups to zeta‐cypermethrin with LC_50_s of 0.64 ppm (95% CI: 0.51–1.34) and 2.82 ppm (95% CI: 1.26–3.63), respectively, although no significant difference between the two. The LC_50_ for MO‐F20 was significantly greater than both Wolfskill and MO‐F1 at 29.67 (95% CI: 24.91–34.21), representing a 46.4 and 10.5‐fold increase, respectively. However, the MO‐F35 population group had no significant difference in LC_50_ value from MO‐F20 at 18.32 due to a very high upper 95% CI (9.098–140.72), resulting in significant overlap with MO‐F20 (Fig. 2). The wide upper CI reflects some extreme estimates in bootstrap resampling due to sparse data in the 12–24 ppm range where the LC_50_ occurs (18.31 ppm); however, the median bootstrap estimate (18.08 ppm) closely matched the estimate, and 75% of bootstrap iterations fell within 16.9–19.7 ppm indicating the estimate is reliable and these few outliers increased the upper range of the 95% CI. Given the similarity in results between males and females, only the female dose–response curve figure is illustrated here. The dose–response curve figure for male analysis is found in the Supporting Information, (Fig. S1).

### 3.2 Population bottleneck

**Figure 3:**
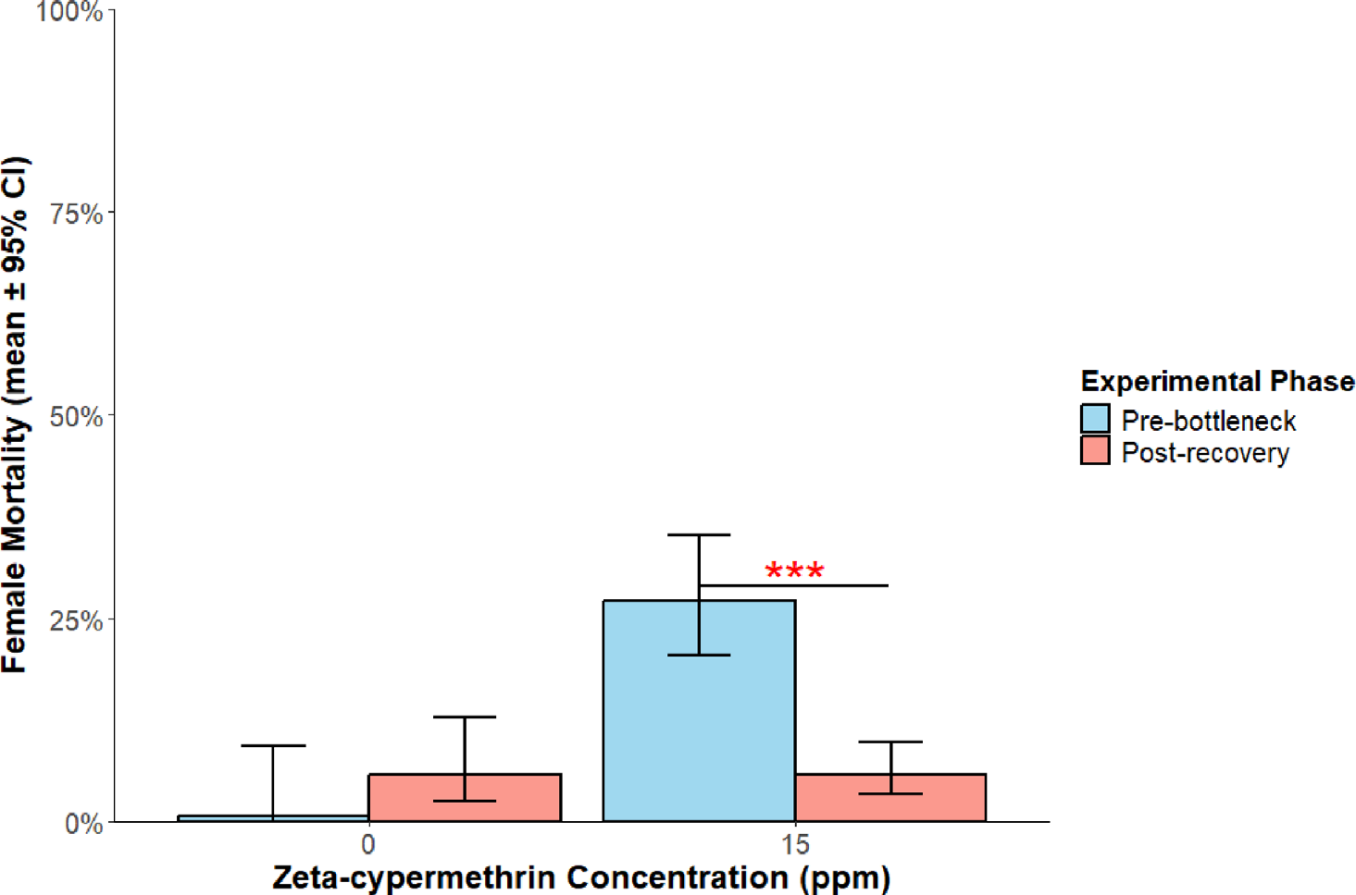
Female mortality (± 95% CI) against zeta‐cypermethrin concentrations both before the population bottleneck event (generations 8–10) and after recovery (generations 19–21) for the untreated controls (M. East untreated line, left) and the treated population (15 ppm, right) with error bars. Red asterisks indicate significant difference at *P* < 0.05.

The LC_50_ values of M. East were recorded at generation F2 and F8, just before the study was commenced, to identify resistance levels prior to treatment. At generation F2 the LC_50_ (baseline resistance for this population) was 126 ppm (approximately 4.2‐fold the label rate when applied at 925 L ha^−1^ (100 gal/ac). Mean female mortality decreased substantially from 27.2% pre‐bottleneck (95% CI: 20.4–35.2) to 5.73% post‐recovery (95% CI: 3.3–9.7%, Fig. 3) during this period. Pre‐bottleneck F8‐10 females had a 6.13‐fold significantly greater odds of death at 15 ppm compared to post‐recovery F19‐21 females (95% CI: 2.7–13.9; *P* < 0.0001). There was also no significant difference between post‐recovery mortality at 15 ppm and the M. East control group mortality at 0 ppm, indicating no effect of exposure to the treatment on mortality than might be expected with natural background levels.

**Figure 4:**
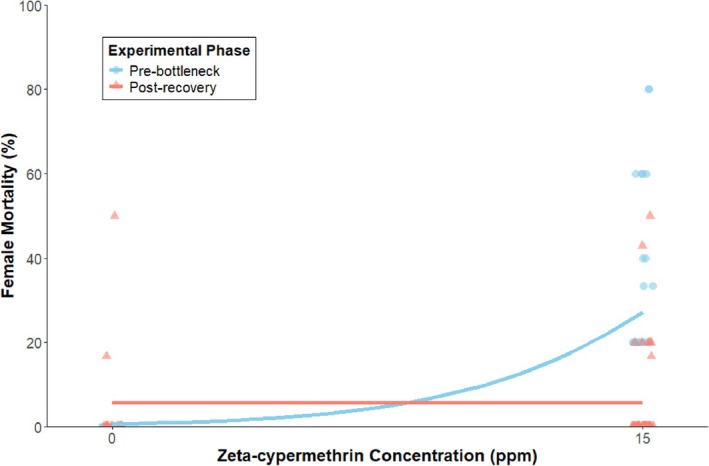
Dose–response curves for female *Drosophila suzukii* mortality both before the population bottleneck event (generations 8–10) and after recovery (generations 19–21) for the untreated controls (0 ppm, left) and the treated population (15 ppm, right).

The dose–response relationship changed significantly between experimental phases, as shown by a significant concentration × phase interaction (β = −0.27 ± 0.10 SE, z = −2.68, *P* < 0.005, Fig. 4). Pre‐bottleneck, the dose–response slope was significantly positive (β = 0.273 ± 0.096 SE, z = 2.84, *P* < 0.005), indicating typical concentration‐dependent mortality where increasing insecticide concentration produced greater mortality. Post‐recovery, the slope was zero (β = −0.001 ± 0.035 SE, z = −0.03, *P* = 0.98) indicating that the two slopes differing significantly (β = 0.274 ± 0.102 SE, z = 2.68, *P* < 0.05). This flat post‐recovery dose–response curve suggests that mortality was independent of concentration across the 0–15 ppm range.

Analysis carried out on males also showed significant declines in mortality in the post‐recovery phase (14.7%, 95% CI: 10.6–20.0%) compared to the pre‐bottleneck phase (31.0%, 95% CI: 23.8–39.3%). However, given that there were no significant differences in the slope of resistance change between sexes, full male analysis and sex comparisons are detailed in the Supporting Information, (S2).

## 4 DISCUSSION

### 4.1 Resistance stability

Results from the dose–response bioassays show that resistance of a tolerant zeta‐cypermethrin population increased when challenged during each generation in a log‐dose manner and then plateaued in the absence of selection after generation 19. The continuous exposure of larvae to the insecticide resulted in greater LC_50_ values in the MOF2O (immediately following selection) and MOF35 (15 generations post selection) populations when compared to the baseline unselected MOF1 and susceptible Wolfskill populations. However, the lack of difference in mortality between MOF20 and MOF35 suggests that the resistance had plateaued at the greater post‐selection concentrations. This highlights that constant selection pressure or exposure to zeta‐cypermethrin treatments or their residues, as commonly occurs on conventionally‐managed farms, may significantly increase resistance in a *D. suzukii* population to a point where the efficacy of zeta‐cypermethrin is significantly reduced and remains stable rather than declines once the selection pressure is removed.

These findings on the build‐up of resistance to commonly used insecticides in *D. suzukii* are supported by studies that carried out similar assessments. For instance, adult exposure to zeta‐cypermethrin for Minnesota populations of *D. suzukii* over a 7–11 month period resulted in a significant increase in resistance, but a more rapid resistance development to spinetoram (a synthetic spinosyn insecticide) was observed.^25^ In another study, larval selection to the insecticides malathion and spinosad over five generations with a sub‐lethal discriminating dose was sufficient to significantly increase resistance to the active ingredients in insecticide tolerant field collected California populations of *D. suzukii* highlighting again the ability for this pest to develop insecticide resistance, especially over a short period of five generations.^13^ Similar results were also observed in field‐collected *D. suzukii* adult females from Georgia, USA that were treated for 10–11 generations with resistance increasing to 7.55‐fold for spinosad and 2.23‐fold for malathion,^26^ as well as in a study on continued selection to spinosad for five generations in a California‐collected population that resulted in significant increases in spinosad resistance.^10^ These results counter findings from a study on *D. suzukii* populations collected in Italian cherry orchards where no resistance selection was achieved using spinosad.^27^ However, it is possible that this population had not yet received sufficient selection for spinosad tolerance in the field at the time lab‐selection was initiated. Conversely, the Civolani *et al*. (2021) study achieved 2.2‐fold increases in LC_50_ values using cyantraniliprole and 25‐fold increases using deltamethrin when compared to the untreated control colony, demonstrating the possibility of resistance development under laboratory selection to active ingredients and therefore posing potential risk for growers using these products. Another study that found no resistance development after continued selection with a sub‐lethal dose used malathion, whereby significant decreases in susceptibility were not observed in a British Columbia population after 30 generations of continued exposure.^28^ As in the case of the Italian spinosad study,^27^ this could be due to low genetic variability in the original population or even microbial degradation of the insecticide. The presence of field‐derived tolerance in populations used in lab resistance studies prior to initiating‐selection may indeed be an important factor influencing results of such studies.

Increasing tolerance in *D. suzukii* populations with continued insecticide applications has also been observed in the field, not only under laboratory‐selection. For example, in 2020, discriminating dose bioassays of field‐captured *D. suzukii* populations from California caneberries showed decreases in susceptibility to the zeta‐cypermethrin as the season progressed alongside continued exposure from grower's treatment programs.^14^ In addition, a field‐collected *D. suzukii* population from Brazil exhibited significantly lower imidacloprid susceptibility when compared with other insecticides such as deltamethrin, spinetoram and permethrin, although this study did not assess resistance development over a sustained period.^15^

The stability of resistance has also been assessed in other pest species, and can exhibit contrary results. For example, in the common vinegar fly, *Drosophila melanogaster* Meigen (Drosophila: Drosophilidae), populations from New York, USA exhibited stable zeta‐cypermethrin and malathion resistance over a period of 33 months in the absence of any treatment, exposure or selection pressure, indicating the potential for resistance development and maintenance in this species as well.^17^ However, resistance in the whitefly, *Bemisia tabaci* Gennadius (Hemiptera: Aleyrodidae) to a variety of insecticides was shown to be unstable when selection pressure was removed.^29^

### 4.2 Population bottleneck and resistance

The results presented here highlight the importance of understanding resistance development and stability during and after a population bottleneck. In our study, the population bottleneck was caused by the doubled dosage of 30 ppm zeta‐cypermethrin used to treat generations 11 and 12 that resulted in greater mortality than anticipated and therefore fewer surviving individuals. Following this bottleneck, mortality to 15 ppm zeta‐cypermethrin was not statistically different from the control group mortality at 0 ppm, suggesting no difference in mortality between natural background mortality of the population that is not exposed to the treatment and a treated, resistant population and, even though no selection pressure was applied for 6 generations. This highlights a potential concern for growers at conventionally managed sites in that even though insecticide applications may result in acceptable mortality for the population at their sites the surviving individuals could replenish a population that would exhibit not only the same but greater levels of resistance than before the bottleneck. These findings represent the first insight into the stability of insecticide resistance in field collected tolerant *D. suzukii* after a population bottleneck in a lab‐controlled environment. Understanding the dynamics causing this could have implications for effective management of the pest in the field. For example, an observed in‐field bottleneck of a Washington state *D. suzukii* population was likely caused by low genetic diversity as well as new introduction into an area which exposed it to environmental factors that could have affected its fitness.^30^ A bottleneck such as this could contribute to a lack of change in susceptibility of insecticide resistance,^28^ or in the case of our study a decrease in susceptibility. This is supported by the theoretical framework presented in a study which states that population dynamics, such as a bottleneck, could alter the outcomes of resistance development due to a genetic drift that could eliminate susceptible alleles.^31^ However, this theoretical possibility depends on the frequency of resistant alleles and duration of the bottleneck. LaBar and Adami (2017) also argue that in small populations (100 or fewer) genetic drift can lead to the further development of resistance if the susceptible alleles exhibit many deleterious mutations, leading the resistant alleles to provide stronger foundations for handling these mutations.^32^ While the study by Labar and Adami (2017) was conducted by modelling digital populations of organisms, the same principles could apply to the bottleneck seen in our study.

Several factors could have affected our results. In a study assessing the mortality of *D. suzukii* when exposed to the insecticide phosmet, younger female adults were more susceptible to the insecticide than females that were a few days older.^33^ In our experiment, all flies tested eclosed within the same 5‐day window, so age could have played a factor as older flies within the cohort exposed to selection may have been less susceptible to mortality after exposure. Other variables could also influence studies such as ours. Insecticide efficacy is known to vary by life stage which may explain why a few studies with different bioassay protocols report results in conflict with our results. Similarly, different types of contact exposure that are better suited for evaluating particular life stages could have an impact.^13^

Fitness costs may also have an effect on resistance stability by selecting against resistance alleles after the removal of exposure to the insecticide.^34^ However, the stability and increase in resistance in our two separate experiments indicates that fitness costs had a minimal effect on the resistance in our *D. suzukii* populations, or the effects of genetic drift outweighed those of the fitness costs.^32^ Endosymbionts such as *Wolbachia* and *Rickettsia* have been shown to aid in the development of insecticide resistance in the tobacco whitefly (*Bemisia tabaci*),^35^ with such mechanisms possibly playing a role in our study. Lastly, zeta‐cypermethrin, although widely used, has been reported to be less reliable in reducing *D. suzukii* numbers through contact bioassays than other insecticides such as phosmet and spinetoram,^36^ so the variability reported for zeta‐cypermethrin by Andika *et al*. (2020)^36^ may have also been present in our study and potentially impacted our results. We recommend further research to focus on resistance stability once the selection pressure is removed not only in field settings but on a wider variety of insecticides as well as this will serve to establish the stability of resistance to different insecticide classes and may help inform *D. suzukii* management practices. We also recommend further research focuses on the stability of resistance during bottlenecks for a more prolonged period and for other insecticides. The resistance levels initially identified at the start of a study might also influence results. Ideally, future research would examine the two population dynamics we present using a range of baseline resistance levels when the studies are initiated. Given that Freeman *et al*. (2021) found 60% of studies exhibit fitness costs influencing resistance stability and this was not the case in ours, we recommend research to further explore these dynamics in *D. suzukii*, which could have implications for pest management programs.

These results also highlight the potential implications for successful management. Firstly, populations should be routinely assessed using discriminating dose or other bioassays which could inform growers on the detection and status of tolerance in their *D. suzukii* populations before resistance becomes obvious and perhaps fixed such that simply not using a chemical for some period would increase the level of susceptibility.^37^ In order to reduce the risk of developing insecticide resistance growers should remove selection pressure by routinely rotating insecticide classes, minimizing prolonged exposure to the same chemical.^38^, ^39^, ^40^

This study is the first to assess not only zeta‐cypermethrin resistance stability in *D. suzukii* following prolonged selection, but also the stability of resistance in the absence of selection pressure and the status of resistance following a population bottleneck. We highlight how readily resistance can develop and how resistance can persist when exposure to the insecticide is removed and how stable the resistance is following a bottleneck.

## Supporting information


**Data S1.** Supporting Information.

## Data Availability

The data that support the findings of this study are available from the corresponding author upon reasonable request.
