# Supplementary material for: Investigating zeta‐cypermethrin resistance stability in California Drosophila suzukii populations
Source: Pest Manag Sci. 2026 Mar 26;82(7):6808–16. doi: 10.1002/ps.70760 (PMC13240691; doi:10.1002/ps.70760)
Supplement: Supplementary file 1 — Data S1. Supporting Information. [file PS-82-6808-s001.docx]

**Supplementary Materials**

1. **Continuous Selection Results**


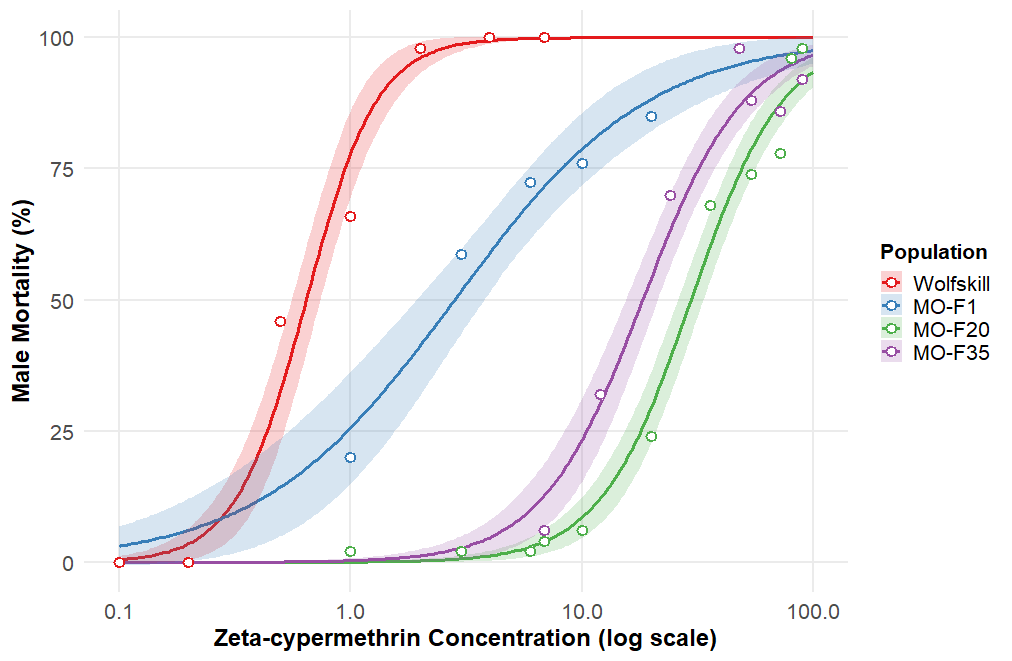


**Figure S1.** Dose-response curves for male Drosophila suzukii exposed to zeta-cypermethrin across a range of concentrations for each experimental group: Wolfskill, and generations 1 (preselection), 20 (immediately after selection termination) and 35 (post selection) of the “MO” population. Solid lines represent fitted two-parameter log-logistic models, with shaded regions indicating 95% confidence intervals (a separate metric from the 95% confidence intervals in the bootstrap resampling). Open circles show observed mortality proportions for individual dose-population combinations. The x-axis presents insecticide concentration on a logarithmic scale (ppm), while the y-axis shows percent mortality.

1. **Bottleneck Male and Sex Comparison Statistics**

Males also showed significant resistance increases following the bottleneck, though with different magnitude than females. Baseline mortality at 0 ppm increased from 1.83% (95% CI: 0.37-8.7%) pre-bottleneck to 11.9% (95% CI: 6.8-20.0%) post-recovery, representing approximately 6.5-fold higher natural mortality. At 15 ppm, mortality decreased from 31.0% (95% CI: 23.8-39.3%) pre-bottleneck to 14.7% (95% CI: 10.6-20.0%) post-recovery, a 52.6% significant reduction (p<0.001). The dose-response slope also changed significantly between phases (p<0.001), confirming substantial resistance evolution in males as well.

Both sexes showed significant resistance increases following the bottleneck event (females: 78.9% reduction, p<0.0001; males: 52.6% reduction, p<0.001). While females showed numerically larger resistance evolution than males (26.3 percentage-point difference), the three-way interaction (sex × phase × concentration) was not statistically significant (β = 0.078 ± 0.119 SE, z = 0.65, p = 0.51). This indicates that while the magnitude of resistance increase differed numerically between sexes, there was a lack sufficient statistical power to conclude that sexes responded differently to the bottleneck.
